# Causal effects of gut microbiome on aortic dissection: A mendelian randomization study

**DOI:** 10.1016/j.clinsp.2025.100811

**Published:** 2025-11-19

**Authors:** Yu Tian, Furong Hao, Guangyang Xu, Yuxuan Qian, Jiahao Liu, Ning Pan, Tao Yang

**Affiliations:** a Vascular Surgery Department, Shanxi Bethune Hospital, Shanxi Academy of Medical Sciences, Third Hospital of Shanxi Medical University, Tongji Shanxi Hospital, Taiyuan, China; b School of Clinical Medicine, Tsinghua University, Beijing, China; c Vascular Department, Beijing Hua Xin Hospital (1st Hospital of Tsinghua University), Beijing, China

**Keywords:** Aortic dissection, Gut microbiome, Mendelian randomization, Causal Inference, FinnGen, Risk factors

## Abstract

**Introduction:**

Aortic Dissection (AD) is a severe vascular disease with high mortality. While its progression is rapid, the development of AD is a chronic disease process. The gut microbiome may play a crucial role in this process. This study aims to explore the potential causal association between gut microbiome and AD using Mendelian Randomization (MR).

**Methods:**

This study employed a two-sample MR approach using Genome-Wide Association Study (GWAS) data from the Finnish population to investigate the causal association between gut microbiota and AD. Single Nucleotide Polymorphisms (SNPs) associated with 473 gut microbiota taxa were selected as instrumental variables, and various MR methods were applied to evaluate the causal effects. Sensitivity analyses were conducted to assess the robustness of the results, and a reverse MR analysis was performed to validate the directionality of the associations.

**Results:**

The MR analysis confirmed that Actinomycetales, Bacillales A, Lawsonibacter sp002161175, Prevotella sp002933775, Saccharomonospora, Acidaminococcus fermentans, CAG-110, CAG-177 sp002451755, CAG-177 sp003514385, and Eisenbergiella sp900066775 may exert protective effects, whereas koll11, Magnetospirillum A, Poseidoniaceae, Pseudomonas aeruginosa, Bacteroides eggerthii, CAG-269 sp002372935, and Dorea phocaeense may increase the risk of AD. Sensitivity analyses confirmed the robustness of these findings.

**Conclusion:**

This study provides new insights into the role of gut microbiome in AD, identifying specific taxa that may serve as protective or risk factors. Further research is needed to validate these findings in broader populations.

## Highlights

• First Mendelian Randomization analysis of gut microbiota and aortic dissection.
• Uses large FinnGen genetic data to minimize confounding and reverse causality.
• Ten protective and eight risk gut microbiome taxa identified for aortic dissection.
• Suggests novel mechanistic pathways involving TMAO, bile acids, and inflammation.
• Shows gut microbiome as a potential therapeutic target for aortic dissection.

## Introduction

Aortic Dissection (AD), the most common form of acute aortic syndrome, is characterized by rapid progression and a high mortality rate, with up to 50 % of cases resulting in death within the first 24 h. Current research on the mechanisms underlying AD development primarily focuses on genetic mutations, vascular wall degeneration, and elastic fiber degradation.^1^^,^^2^ However, despite the acute onset of AD, its progression is chronic, and effective strategies to manage or prevent its chronic development remain unclear. Current treatments mainly focus on addressing the immediate acute events, with little emphasis on the long-term management of vascular health in AD patients. This gap highlights the urgent need for novel approaches to manage AD's chronic progression.

The gut microbiome, particularly bacterial communities, plays a significant role in the management and prevention of disease development, and has been notably associated with the development of cardiovascular diseases.^3^, ^4^, ^5^ Through their metabolic products, the gut microbiome can influence host metabolism and immune function, serving not only to maintain normal physiological functions but also acting as regulators in the pathogenesis of vascular diseases. Given these key roles, the authors hypothesize that investigating the role of gut microbiome in AD could offer a novel scientific basis for developing future prevention and management strategies for AD.

Mendelian Randomization (MR) is a rigorous research method that leverages naturally occurring genetic variations as instrumental variables to provide an unbiased estimate of the potential causal effects of specific exposures on health outcomes. The strength of this approach lies in the inherent determinism of genetic variations and their random distribution within populations, akin to the design principles of randomized controlled trials. This characteristic effectively minimizes confounding factors, thereby enhancing the precision of causal inference.^6^

Given its advantages, MR has demonstrated broad applicability in medical research,^7^ with significant value in cardiovascular disease research. For example, MR has revealed causal links between gut microbiota and peripheral artery disease^8^ as well as between lipid levels and coronary heart disease,^9^ highlighting its utility in understanding vascular disease mechanisms. This study applies the MR approach to explore the potential causal association between the gut microbiome and AD, a severe vascular condition. By utilizing this method, the authors aim to uncover novel associations, providing robust data and fresh insights that could inform the development of prevention and therapeutic strategies for AD, ultimately contributing to better disease management.

## Method

### Study design and the three assumptions of MR

**Fig. 1:**
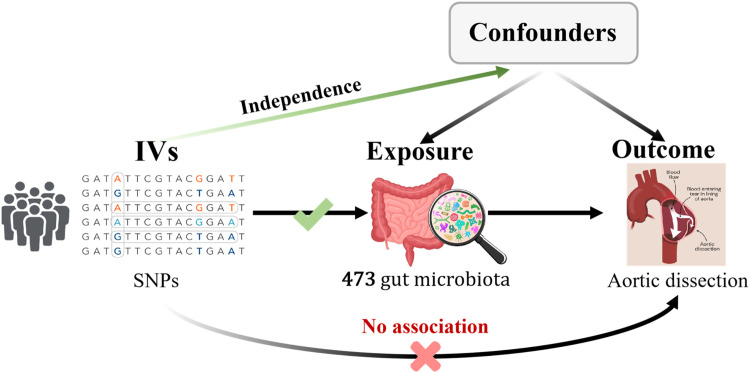
The three core assumptions of Mendelian Randomization (MR).

This study is documented in accordance with the guidelines set forth in the Strengthening the Reporting of Observational Studies in Epidemiology Using MR (STROBE-MR, S1 Checklist) as recommended by Skrivankova et al.^10^ By utilizing a two-sample MR methodology, the authors investigated the causal link between the gut microbiota and AD. The MR analysis is predicated upon three core assumptions, as depicted in Fig. 1: 1) The Instrumental Variables (IVs) chosen from the dataset must exhibit a strong correlation with the exposure; 2) IVs should not influence the outcome through any route other than the exposure, indicating they must be free from confounding factors; 3) The influence of IVs on the outcome must be mediated exclusively through the exposure and not through alternative pathways. This research explores the causal impact of particular gut microbiota on AD, designating gut microbiota as the exposure and AD as the outcome. A bidirectional MR approach is employed to verify the directionality of the causal association, with AD as the exposure and gut microbiota as the outcome.

### Data sources and ethics statement

This study employed an MR approach, based on data obtained from publicly available Genome-Wide Association Studies (GWAS). Since the data were derived from previously published studies, all original data had received ethical approval, and informed consent had been obtained by the original researchers. Specifically, data related to the gut microbiota were sourced from the FINRISK 2002 cohort, which includes shallow shotgun metagenomic sequencing data from 7231 samples covering 478 gut microbiota taxa from 5959 individuals aged 25 to 74 years, originating from six geographical regions of Finland.^11^ The complete summary statistics for these microbial taxa, including genome-wide significant hits, are publicly available in the NHGRI-EBI GWAS Catalog (https://www.ebi.ac.uk/gwas/) under accession numbers GCST90032172 to GCST90032644. The statistical data on AD were obtained from the Finnish database’s global study (pheno I9_AORTDIS at finngen.fi),^12^ which includes 967 AD patients and 381,977 healthy controls. The patient group comprised 244 females and 723 males, with a mean age of onset of 65.09 years.

### Selection of IVs

IVs were identified through the precise localization of SNPs associated with the gut microbiota. To maintain the accuracy and validity of the inferred causal association between gut microbiota composition and AD, the following criteria were established for IV selection: 1) Since loci associated with gut microbiota rarely achieve the conventional genome-wide significance level (*p* < 5 × 10⁻⁸) as identified in GWAS, SNPs linked to gut microbiota with a *p* < 1 × 10^−5^ were selected as candidate IVs.^13^, ^14^, ^15^, ^16^ 2) Reference panels constructed from European samples in the 1000 Genomes Project were used to assess Linkage Disequilibrium (LD) among the SNPs. SNPs with an R^2^ below 0.001 within a clumping window of 10,000 kb were prioritized based on the most significant p-values. 3) SNPs with a Minor Allele Frequency (MAF) of 0.01 or less were excluded from further consideration. 4) To align SNP effects on both the exposure and the outcome, palindromic SNPs were excluded, and strand and allele inconsistencies were corrected according to the human genome reference (build 37). 5) The reliability of each SNP was evaluated using the F-statistic, $F=\frac{R^{2}}{1-R^{2}}.\frac{N-K-1}{K}$,where R^2^ indicates the proportion of variance in the exposure explained by genetic variants, N is the sample size, and K represents the number of instrumental variables. SNPs exhibiting an F-statistic of 10 or higher were considered sufficiently reliable, whereas those with a lower F-statistic were deemed inadequate and excluded. Furthermore, the independence of the selected IVs was ensured by verifying against the ldlink.nih.gov/?tab=ldtrait website,^17^ eliminating any potential confounding SNPs that might have a causal association with AD.

In reverse MR studies, where AD was treated as the exposure and the gut microbiota as the outcome, the selection criterion for IVs was set at *p* < 5 × 10^−8^, with other methods applied similarly.

### Statistical analysis

#### MR analysis

In this study, the objective was to elucidate the causal association between gut microbiota and AD through the application of several validated MR techniques. The methods employed included the Inverse Variance Weighted (IVW) approach, weighted mode, MR-Egger regression, weighted median estimator, and simple mode. Among these, the IVW method served as the primary analytical tool, especially in scenarios involving multiple instrumental variables.^10^ The integration of these varied methodologies enabled a more thorough and nuanced exploration of the hypothesized causal connections.

#### Sensitivity analysis

Beyond the primary MR analyses, the authors conducted extensive sensitivity analyses to ascertain the robustness and credibility of the present findings. This included using Cochran's *Q* statistics to evaluate heterogeneity, with statistical significance determined by a threshold of *p* < 0.05. The Steiger test was also employed to confirm the relevance of the instrumental variables, ensuring they predominantly affected the exposure rather than the outcome, thereby reinforcing proposed causal direction. Additionally, the MR-Presso global test and MR-Egger regression were applied to detect and address potential pleiotropy within the dataset. To further affirm the stability and reliability of these conclusions, a leave-one-out analysis was performed.

#### Analytical software

All statistical analyses were executed using *R* software, version 4.3.3, developed by The *R* Foundation for Statistical Computing, Vienna, Austria. Specifically, the *R* packages Two Sample MR (version 0.5.7) and MR-PRESSO (version 1.0) were utilized to perform the MR analyses.

## Results

**Fig. 2:**
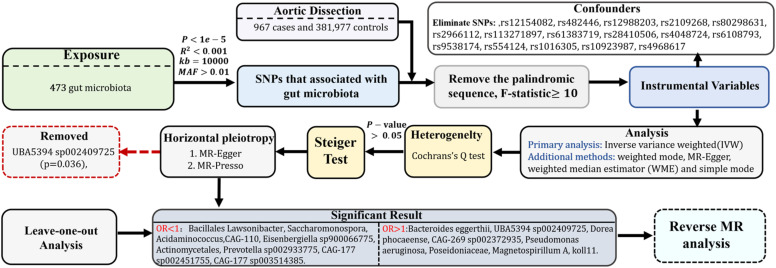
Flowchart of the study.

The comprehensive process of this research is depicted in Fig. 2.

### Selection and characteristics of IVs

Following the selection criteria for IVs, the authors initially identified a set of potential IVs. Subsequently, through a comprehensive review of epidemiological literature and by incorporating confounding factor data from the LDlink website, the authors excluded SNPs affected by confounders such as hypertension and smoking,^18^^,^^19^ including rs12154082, rs482446, rs12988203, rs2109268, rs80298631, rs2966112, rs113271897, rs61383719, rs28410506, rs4048724, rs6108793, rs9538174, rs554124, rs1016305, rs10923987, and rs4968617. Ultimately, the authors identified 7789 SNPs that met the *p* < 1 × 10^−5^ threshold, which were selected as the final IVs. Detailed information on all SNPs is provided in Table S1.

### Main mendelian randomization results

The authors analyzed the causal effects between 473 gut microbiota taxa, derived from the Finnish population, as exposure factors and AD as the outcome. Detailed results are presented in Table S2, which includes information on the results of five MR analysis methods and the number of SNPs. Using the IVW method as the primary approach for MR analysis, 18 gut microbiota taxa were identified as having significant causal associations with AD. The study found that the following taxa exhibited protective effects against AD: Actinomycetales (OR = 0.552, 95 % CI 0.3380–0.900, *p* = 0.017), Bacillales A (OR = 0.024, 95 % CI: 0.0013–0.463, *p* = 0.013), *Lawsonibacter sp002161175* (OR = 0.264, 95 % CI 0.1122–0.621, *p* = 0.002), *Prevotella sp002933775* (OR = 0.588, 95 % CI: 0.3490–0.991, *p* = 0.046), *Saccharomonospora* (OR = 0.423, 95 % CI: 0.1882–0.952, *p* = 0.038), *Acidaminococcus fermentans* (OR = 0.544, 95 % CI: 0.3277–0.904, *p* = 0.019), *CAG-110* (OR = 0.546, 95 % CI 0.3223–0.925, *p* = 0.024), *CAG-177 sp002451755* (OR = 0.663, 95 % CI 0.4579–0.961, *p* = 0.030), *CAG-177 sp003514385* (OR = 0.724, 95 % CI 0.5630–0.931, *p* = 0.012), and *Eisenbergiella sp900066775* (OR = 0.551, 95 % CI 0.3289–0.924, *p* = 0.024).

Conversely, the following taxa were associated with an increased risk of AD: *koll11* (OR = 7.520, 95 % CI 1.1950–47.323, *p* = 0.032), *Magnetospirillum A* (OR = 5.006, 95 % CI 1.2091–20.730, *p* = 0.026), *Poseidoniaceae* (OR = 4.964, 95 % CI 1.0981–22.440, *p* = 0.037), *Pseudomonas aeruginosa* (OR = 3.233, 95 % CI 1.1282–9.265, *p* = 0.029), *Bacteroides eggerthii* (OR = 1.294, 95 % CI: 1.0171–1.646, *p* = 0.036), *UBA5394 sp002409725* (OR = 1.657, 95 % CI 1.0200–2.693, *p* = 0.041), *CAG-269 sp002372935* (OR = 2.172, 95 % CI 1.3033–3.619, *p* = 0.003), and *Dorea phocaeense* (OR = 2.028, 95 % CI 1.0600–3.881, *p* = 0.033).

**Fig. 3:**
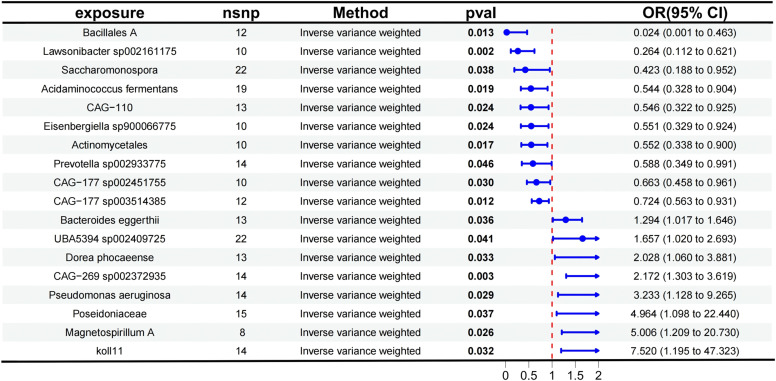
Forest plot showing the results of the Mendelian Randomization (MR) analysis evaluating the association between gut microbiota and aortic dissection using the Inverse Variance Weighted (IVW) method. The effect size (OR, 95 % CI) and statistical significance (p-value < 0.05) for each gut microbiota are displayed.

**Fig. 4:**
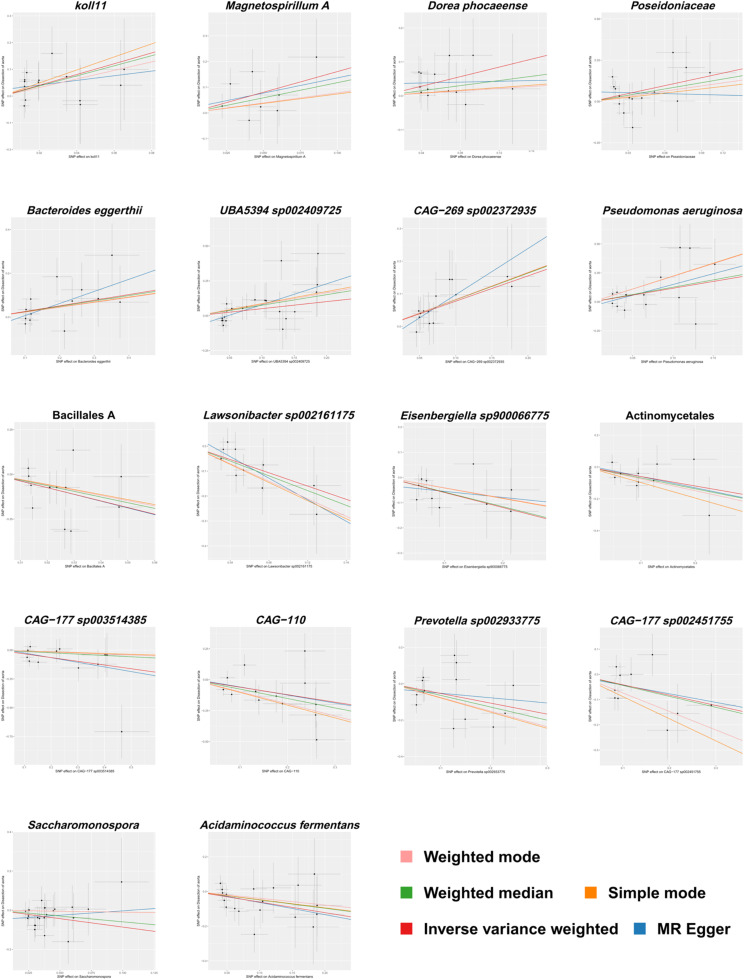
Scatter plots illustrating the causal association between gut microbiota and aortic dissection as evaluated by five different Mendelian Randomization (MR) methods. Each curve represents one MR method, and each point reflects the effect estimate of an individual SNP.

The aforementioned results are presented in Fig. 3. Fig. 4 provides a visual representation of the preliminary analysis of the association between 18 gut microbiota and AD, using five different MR methods. Detailed quantitative results and statistical significance analyses can be found in Table S3. The SNP details for these 18 significant gut microbiotas are provided in Table S4.

### Sensitivity analysis

**Fig. 5:**
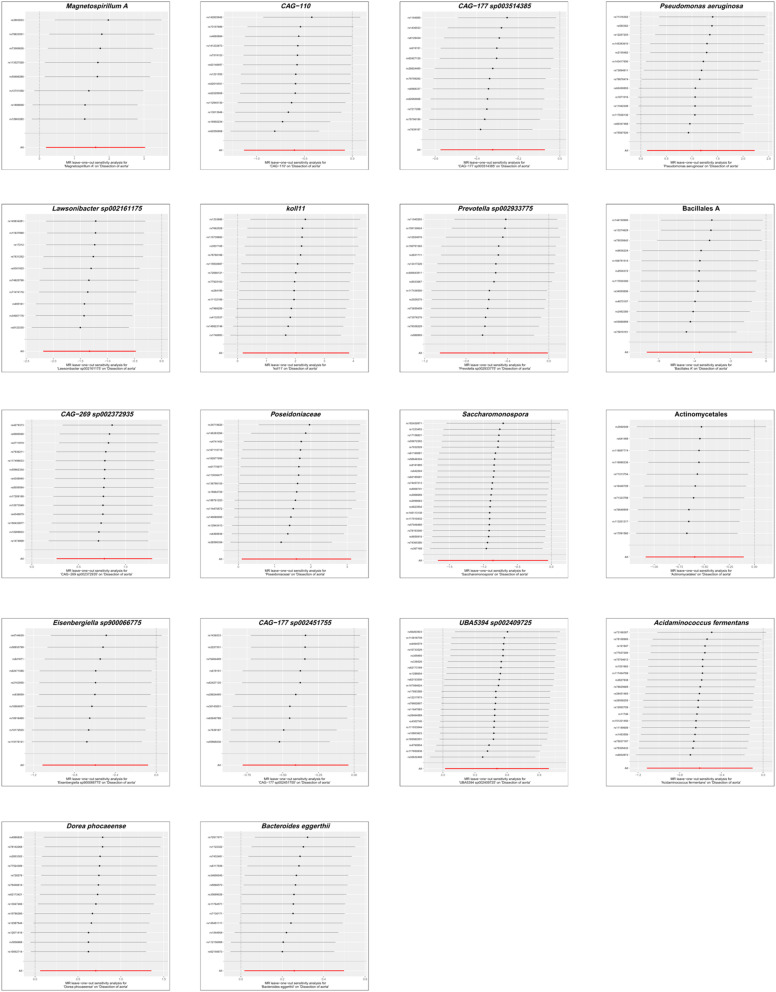
Leave-one-out analysis showing the impact of each Single Nucleotide Polymorphism (SNP) on the causal association between gut microbiota and aortic dissection in the Mendelian randomization analysis. Each point represents the recalculated effect estimate after removing a specific SNP, with the X-axis showing the effect estimate and the Y-axis representing the SNP identifier. The red line indicates the overall effect estimate.

To evaluate the heterogeneity present within the present dataset, the authors applied Cochran’s *Q* statistic to the positive outcomes of the IVW analysis, with all corresponding p-values exceeding 0.05, which demonstrates a lack of significant heterogeneity among the Instrumental Variables (IVs). These results, along with the outcomes of the Cochran’s *Q* test, are comprehensively summarized in Table 1. The Steiger test, also detailed in Table 1, validated the directionality of the IVs, confirming that their influence on the gut microbiota was considerably stronger than their direct effects on AD. The MR-Presso global test further reinforced these findings, with all p-values associated with positive MR analyses of gut microbiota exceeding 0.05, as shown in Table 1. The MR-Egger regression, as presented in Table 1, identified horizontal pleiotropy in the UBA5394 sp002409725 (*p* = 0.036), leading to its exclusion from the final causal analysis between gut microbiota and AD. The other MR-Egger regression results had p-values above 0.05, and are also listed in Table 1. Subsequently, the leave-one-out analysis plots (Fig. 5) indicated that there were no significant outliers among the instrumental variables contributing to the positive results, further supporting the findings presented in Table 1.

**Table 1:** Sensitivity analyses for causality from gut microbiota on aortic dissection.

Exposure	Cochran’s *Q*	Cochran’s *Q* p-value	Steiger p-value	MR-Egger Intercept	MR-Egger Intercept p-value	MR-Presso Global p-value
koll11	7.875	0.852	3.00E-66	0.024	0.501	0.885
Actinomycetales	4.505	0.875	3.12E-72	0.011	0.849	0.911
Bacillales A	18.071	0.080	1.61E-58	0.001	0.986	0.107
Lawsonibacter sp002161175	4.119	0.903	1.88E-46	0.045	0.414	0.921
Magnetospirillum A	4.849	0.678	1.37E-37	0.018	0.805	0.697
Poseidoniaceae	17.301	0.240	1.30E-73	0.059	0.235	0.285
Prevotella sp002933775	18.248	0.148	2.13E-65	−0.027	0.609	0.155
Pseudomonas aeruginosa	16.549	0.221	1.51E-64	−0.036	0.448	0.246
Saccharomonospora	10.681	0.969	7.35E-109	−0.048	0.329	0.971
Bacteroides eggerthii	10.401	0.581	8.64E-60	−0.048	0.298	0.606
UBA5394 sp002409725	21.575	0.424	3.10E-108	−0.071	0.036	0.432
Acidaminococcus fermentans	12.396	0.826	5.75E-87	0.007	0.846	0.845
CAG-110	20.414	0.060	7.07E-59	0.006	0.936	0.064
CAG-177 sp002451755	11.173	0.264	9.79E-48	−0.008	0.892	0.312
CAG-177 sp003514385	13.164	0.283	8.21E-56	0.015	0.770	0.321
CAG-269 sp002372935	3.841	0.993	3.65E-63	−0.044	0.402	0.998
Dorea phocaeense	4.567	0.971	3.52E-62	0.035	0.521	0.976
Eisenbergiella sp900066775	4.421	0.882	6.35E-47	−0.031	0.552	0.903

### Reverse mendelian randomization analysis

Based on the results of the forward MR analysis, the authors conducted a reverse MR study to assess the robustness of the causal inference. In this analysis, AD was used as the exposure, while the aforementioned 18 gut microbiota taxa served as the outcomes. The MR analysis was performed with a significance threshold of *p* < 5 × 10^−8^, as detailed in Table S5. A more relaxed threshold of *p* < 1 × 10^−5^, consistent with that used in the forward MR analysis, was also applied in the reverse MR analysis, as detailed in Table S6. The results from IVW of MR methods indicated that the occurrence of AD does not affect the abundance of these microbial taxa.

## Discussion

Our study is based on data from multiple Finnish cohorts, which share similar environmental factors such as diet, healthcare access, and socioeconomic status. These factors significantly influence the relationship between exposure and outcome. Studying a population within a relatively uniform environment helps control for these variables, thereby reducing confounding effects and enhancing the precision of causal inference. Furthermore, the relatively homogeneous genetic background within a single country minimizes genetic heterogeneity, further strengthening the reliability of causal estimates.^12^

To our knowledge, this is the first study to suggest a potential causal association between gut microbiome and AD using MR. Through this analysis, the authors effectively eliminated potential confounders and pleiotropic biases, ultimately identifying and confirming the associations of Actinomycetales, Bacillales A, *Lawsonibacter sp002161175*, Prevotella sp002933775, *Saccharomonospora, Acidaminococcus fermentans, CAG-110, CAG-177 sp002451755, CAG-177 sp003514385, Eisenbergiella sp900066775, koll11, Magnetospirillum A, Poseidoniaceae, Pseudomonas aeruginosa, Bacteroides eggerthii, CAG-269 sp00237–2935 and Dorea phocaeense* with AD. These findings not only broaden the understanding of the pathological mechanisms underlying AD but also provide new insights and potential strategies for preventing the disease through modulation of the gut microbiome.

The development and progression of AD involve multiple pathophysiological mechanisms, including but not limited to the persistent effects of hypertension, Endothelial Cell (EC) dysfunction, Vascular Smooth Muscle Cell (VSMC) phenotypic transformation, enhanced oxidative stress, and complex inflammatory regulation.^20^

Hypertension is a significant risk factor for AD. Prolonged exposure to elevated blood pressure increases shear stress on blood vessels, leading to enhanced vascular wall stiffness, which can ultimately trigger AD. Notably, both hypertensive and AD patients exhibit strikingly similar gut microbiome diversity and composition.^21^ Specifically, these groups show reduced microbial richness and a marked decrease in beneficial bacterial populations. Glycine, a gut-derived metabolite, has garnered considerable attention due to its anti-inflammatory and antioxidant properties. Studies have indicated that individuals with higher glycine levels tend to have a lower risk of cardiovascular diseases such as hypertension. Further analysis revealed a significant positive correlation between glycine levels and the abundance of Actinomycetales and *Prevotella*.^22^^,^^23^ This may indirectly support a link between Actinomycetales and *Prevotella* and aortic dissection, consistent with the positive associations identified in the present study.

Endothelial dysfunction is influenced by multiple factors, with Trimethylamine N—Oxide (TMAO) emerging as a key player in clinical studies, where it has been confirmed to induce EC dysfunction and accelerate AD progression. TMAO levels are positively correlated with the severity of AD, a finding supported by numerous studies.^24^^,^^25^ Specifically, AD patients exhibit significantly elevated TMAO levels compared to healthy individuals, especially during the hyperacute phase of the disease. Notably, there is no significant difference in TMAO levels across different AD phenotypes, suggesting that elevated TMAO is a common feature in AD progression. Previous studies have suggested that inhibiting TMAO production or reducing its levels may help mitigate the pathological progression of AD.^26^ Importantly, gut microbiome composition is closely linked to TMAO levels. Specifically, an increase in the relative abundance of *Dorea* and *Bacteroides eggerthii* is positively correlated with elevated TMAO levels, suggesting a possible role of gut microbiome in regulating TMAO levels and AD risk.^26^^,^^27^ Conversely, when TMAO levels rise, the abundance of *Prevotella* decreases, aligning with these findings that *Prevotella* may exert a protective effect against AD, while *Dorea* is considered a risk factor.^28^ Additionally, Indole-3-Propionic Acid (IPA), a gut microbial metabolite, has shown strong anti-inflammatory properties and the ability to reduce EC apoptosis. These characteristics may be important in modulating early EC dysfunction in AD and are significantly negatively correlated with AD risk.^29^ Of particular importance, oral IPA has been shown to reduce the abundance of *Bacteroides* species,^30^ which may be consistent with the observation that *Bacteroides eggerthii* is a potential risk factor for AD.

In the pathogenesis of AD, abnormalities in aortic structure and function are central, with the contractile regulation of VSMC playing a critical role in maintaining the overall architecture and physiological integrity of the aorta. Recent studies have demonstrated that Indole-3-Aldehyde (3-IAID), a bioactive molecule, effectively inhibits VSMC phenotypic transformation, providing new strategic support for slowing aortic degenerative changes.^31^ Research has confirmed that Actinomycota is a natural producer of 3-IAID,^32^ while *Pseudomonas aeruginosa* has shown the ability to degrade 3-IAID.^33^ These findings are consistent with this conclusion that Actinomycetales may have a protective effect, while *Pseudomonas aeruginosa* may induce adverse effects, further emphasizing the role of gut microbiome in aortic health. Moreover, the application of Ursodeoxycholic Acid (UDCA) has received extensive attention in the study of VSMC protection mechanisms. Experimental data indicate that UDCA significantly inhibits angiotensin II-induced VSMC apoptosis.^34^ Additionally, animal model studies have reported a significant positive correlation between CAG-110, Prevotella, and bile acid levels.^35^ These findings expand current understanding of the involvement of these microbes in bile acid metabolism. The present observations that CAG-110 and Prevotella may serve as protective factors are consistent with these reports, highlighting the complex role of the gut microbiome in aortic diseases.

In the exploration of AD pathogenesis, the inflammatory response plays a pivotal role in the chronic erosion of the vascular wall. Scientific research has clearly demonstrated that AD patients have significantly elevated levels of inflammatory markers compared to healthy individuals, with Reactive Oxygen Species (ROS) and inflammatory factor surges being recognized as two core mechanisms of AD progression.^21^^,^^36^ 3-IAID, as a potential therapeutic strategy, exhibits the ability to reduce inflammatory factor infiltration into the vascular wall and inhibit the inflammatory response within the aortic wall, showing promise in effectively preventing AD formation and progression.^31^ These findings may provide insights into the distinct roles that Actinomycota and *Pseudomonas aeruginosa* may be involved in during AD pathogenesis. Macrophages, as key regulators of vascular wall inflammation, exacerbate the cascade of local inflammatory responses by secreting inflammatory factors. Specifically, succinate accumulation within macrophages has been shown to trigger the production of large amounts of ROS, a process that is positively correlated with the exacerbation of aortic disease.^37^ The production of succinate is primarily regulated by bacteria such as *Bacteroides*, while its consumption largely depends on the activity of Acidaminococcaceae.^38^ Moreover, Jiang et al.^21^ explored the phenomenon of Bacteroidota enrichment in AD patients and proposed that this enrichment may influence the body's sensitivity to inflammatory stimuli, providing a new perspective on the role of microbiota in the pathogenesis of AD. On the other hand, *CAG-110* and *Prevotella* have shown the ability to inhibit ROS production by regulating bile acid metabolic pathways.^34^ Notably, *Prevotella* has been found to mitigate the inflammatory response induced by *Pseudomonas aeruginosa*.^39^ This discovery underscores the importance of microbial interactions in regulating the inflammatory response. In summary, these findings suggest that *Bacteroides eggerthii* and *Pseudomonas aeruginosa* may be risk factors for AD, whereas *Prevotella* and *Acidaminococcus fermentans* (belonging to the family Acidaminococcaceae) may serve protective roles, consistent with current evidence.

While the present study offers significant strengths, certain limitations should be acknowledged. First, this analysis is based on data from the Finnish population. Due to differences in diet, genetics, and lifestyle, gut microbiota composition varies significantly across populations, which may limit the generalizability of these findings, particularly to populations with distinct genetic backgrounds and lifestyles, such as Asian or African cohorts. Therefore, future studies need to be conducted in more diverse populations to validate the generalizability of these findings, with priority given to replication in multi-ethnic GWAS datasets. Second, although MR is a powerful tool for causal inference, potential residual horizontal pleiotropy and unmeasured confounding factors cannot be entirely excluded. Though sensitivity analyses mitigate this concern, some degree of pleiotropy remains possible. Third, while the present study identifies specific gut microbiota taxa associated with AD risk, the underlying biological mechanisms remain unclear. Further experimental and functional studies are needed to elucidate how these microbial taxa contribute to AD pathogenesis. Lastly, this study relies on GWAS data, which primarily captures host genetic influences on gut microbiome and AD risk. The absence of longitudinal data limits the ability to assess the dynamic interactions between gut microbiome, environmental exposures, and AD progression.

Nevertheless, this study possesses several notable strengths. To our knowledge, it is the first to establish a causal association between gut microbiome and AD using MR, addressing a significant gap in the literature. By leveraging large-scale, well-characterized datasets ‒ including gut microbiome profiles from 5959 individuals and a GWAS of 967 AD cases with 381,977 controls ‒ the authors ensured robust statistical power. Furthermore, this study comprehensively investigates 473 gut microbial taxa, providing unprecedented insights into the gut microbiota-AD relationship. These strengths enhance the robustness of these conclusions and underscore the need for further research to validate and expand upon the present findings.

From a clinical perspective, these findings highlight the gut microbiome as a promising avenue for further investigation in AD research. Although interventions such as probiotics, prebiotics, or dietary modifications may theoretically influence the microbial taxa implicated in this study, these approaches remain speculative at this stage. Notably, strategies targeting gut microbial metabolites (e.g., TMAO) have shown potential in related cardiovascular diseases, suggesting possible translational value.^40^ However, further functional studies, such as fecal microbiota transplantation experiments in animal models, are necessary to validate the mechanistic roles of specific microbes in AD pathogenesis. Moreover, clinical trials will be crucial to advance these foundational findings toward clinical application. In summary, the present results provide genetic evidence supporting the exploration of gut microbiota-targeted interventions as hypotheses for future research.

## Conclusion

In conclusion, this MR study provided novel evidence of a potential causal association between gut microbiome and AD in the Finnish population. The authors identified 18 specific microbial taxa linked with AD risk – ten taxa (e.g., Actinomycetales, Bacillales A, *Prevotella sp002933775*, etc.) that may exert protective effects, whereas eight taxa (e.g., *Pseudomonas aeruginosa, Bacteroides eggerthii, Dorea phocaeense*, etc.) that may increase the risk of AD. These results offer new insights into the pathophysiological mechanisms underlying AD and highlight the critical role of the gut microbiome in this disease. Although the present study is based on data from a Finnish cohort, the findings provide a crucial scientific basis for further research into the role of the gut microbiome in AD. Future studies are warranted to validate these associations in more diverse populations and to explore the specific biological mechanisms by which these microbes influence the onset and progression of AD.

## Authors’ contributions

Conception and design: YT, FH, TY. Analysis and interpretation: YT, FH, GX, YQ, JL, NP, TY. Data collection: YT, JL. Writing the article: YT, FH, GX, GX, YQ. Critical revision of the article: YT, FH, GX, YQ, JL, NP, TY. Final approval of the article: YT, FH, GX, YQ, JL, NP, TY. Statistical analysis: YT, FH, GX, YQ, JL. Obtained funding: YT, TY. Overall responsibility: TY. YT and FH contributed equally to this article and share co-first authorship.

## Funding

This work was supported by the Fundamental Research Program of Shanxi Province (202303021222321) and the Scientific foundation of Shanxi Health Commission (2020003).

## Declarations

Declaration on the Use of Generative AI and AI-Assisted Technologies in the Writing of This Paper. While composing this study, the authors employed ChatGPT to improve the manuscript's language and overall quality. The refined version of the manuscript has been thoroughly reviewed and revised by the authors, who take full responsibility for the content that has been published.

## Ethics committee information and study protocol

Ethics Committee study protocol number: Not applicable.

This study utilized publicly available GWAS data and did not involve direct patient recruitment or data collection. Ethical approval for the original data collection was granted by the respective Ethics Committees of the institutions involved. As the data used in this study were publicly available and anonymized, no new ethics approval or consent was required. The original study protocols were approved by the relevant ethics committees, and informed consent was obtained from all participants involved in the primary studies.

## Consent for publication

All authors have approved the final version of the manuscript and have given their consent for its submission for publication in this journal.

## Data availability

The datasets utilized in this study are derived from publicly accessible databases, including the FINRISK 2002 cohort study and the FinnGen project. These resources provide researchers with access to the relevant data. Detailed information regarding data access can be found on the official GWAS Catalog website (accession numbers: GCST90032172 to GCST90032644) as well as on the FinnGen project’s official website.

## Declaration of competing interest

The authors declare no conflicts of interest.

## Appendix Supplementary materials

Image, application 1
